# A Preliminary Genome-Wide Association Study of Pain-Related Fear: Implications for Orofacial Pain

**DOI:** 10.1155/2017/7375468

**Published:** 2017-06-15

**Authors:** Cameron L. Randall, Casey D. Wright, Jonathan M. Chernus, Daniel W. McNeil, Eleanor Feingold, Richard J. Crout, Katherine Neiswanger, Robert J. Weyant, John R. Shaffer, Mary L. Marazita

**Affiliations:** ^1^Department of Psychology, West Virginia University, 53 Campus Drive, P.O. Box 6040, Morgantown, WV 26506, USA; ^2^Department of Dental Practice & Rural Health, Center for Oral Health Research in Appalachia (COHRA), School of Dentistry, West Virginia University, One Medical Center Drive, Morgantown, WV 26506, USA; ^3^Department of Human Genetics, Graduate School of Public Health, University of Pittsburgh, 130 De Soto Street, Pittsburgh, PA 15261, USA; ^4^Center for Oral Health Research in Appalachia (COHRA), University of Pittsburgh, Pittsburgh, PA 15260, USA; ^5^Department of Periodontics, Center for Oral Health Research in Appalachia (COHRA), School of Dentistry, West Virginia University, One Medical Center Drive, Morgantown, WV 26506, USA; ^6^Department of Oral Biology, Center for Craniofacial and Dental Genetics, School of Dental Medicine, University of Pittsburgh, Bridgeside Point Suite 500, 100 Technology Drive, Pittsburgh, PA 15219, USA; ^7^Department of Dental Public Health, School of Dental Medicine, University of Pittsburgh, 3501 Terrace Street, Pittsburgh, PA 15261, USA

## Abstract

**Background:**

Acute and chronic orofacial pain can significantly impact overall health and functioning. Associations between fear of pain and the experience of orofacial pain are well-documented, and environmental, behavioral, and cognitive components of fear of pain have been elucidated. Little is known, however, regarding the specific genes contributing to fear of pain.

**Methods:**

A genome-wide association study (GWAS; *N* = 990) was performed to identify plausible genes that may predispose individuals to various levels of fear of pain. The total score and three subscales (fear of minor, severe, and medical/dental pain) of the Fear of Pain Questionnaire-9 (FPQ-9) were modeled in a variance components modeling framework to test for genetic association with 8.5 M genetic variants across the genome, while adjusting for sex, age, education, and income.

**Results:**

Three genetic loci were significantly associated with fear of minor pain (8q24.13, 8p21.2, and 6q26; *p* < 5 × 10^−8^ for all) near the genes* TMEM65*,* NEFM*,* NEFL*,* AGPAT4*, and* PARK2*. Other suggestive loci were found for the fear of pain total score and each of the FPQ-9 subscales.

**Conclusions:**

Multiple genes were identified as possible candidates contributing to fear of pain. The findings may have implications for understanding and treating chronic orofacial pain.

## 1. Introduction

The experience of orofacial pain is distressing, with burden observed at individual and societal levels. For instance, people experiencing orofacial pain are likely to miss work, have impaired sleep, and have poorer quality of life [1–3]. At a societal level, orofacial pain is estimated to cost the United States over $4 billion annually, a product of healthcare expense, disability, and lost productivity [4]. Orofacial pain can be experienced acutely, as in the case of toothache or pain related to a dental procedure, and/or chronically, such as in temporomandibular joint disorder (TMD) or trigeminal neuralgia [5]. The experience of acute orofacial pain is universal, and an estimated one-quarter to one-third of people will suffer from chronic orofacial pain during their lifetime [6, 7]. Thus, orofacial pain represents a relatively prevalent problem and a particularly important one given its potential to impact health and functioning. To most effectively address the problem of orofacial pain, it is necessary to develop a comprehensive model of its etiology, which requires a nuanced understanding of a range of contributing factors.

It is well established that the experience of pain, including orofacial pain, is impacted to a large degree by psychological phenomena. In the case of acute orofacial pain, for example, the presence of moderate-to-high levels of fear is known to intensify the pain experience, with strong associations observed between fear and pain threshold and tolerance [8–10]. Likewise, depression, anxiety, and history of posttraumatic stress disorder are associated with more intense, functionally limiting, and/or treatment-resistant chronic orofacial pain [11–14]. Indeed, chronic pain conditions, including chronic orofacial pain, are considered to have both physical and psychological etiologies, with emotion and other psychological variables frequently contributing to maintenance of the condition [14–16].

One psychological phenomenon that is particularly important in the experience of pain is fear of pain, which is defined as dread of or apprehension about nociception associated with environmental experiences or bodily phenomena that cause pain [17, 18]. At high levels, fear of pain can be irrational and maladaptive, negatively influencing behavior and contributing to the maintenance of chronic pain symptomatology via a pain-avoidance cycle [17]. For example, fear of chronic orofacial pain can develop when an individual is apprehensive about the recurrence of acute orofacial pain flares or certain mandibular movements (e.g., chewing of certain foods). Avoidance and emotional distress can continue, however, even after the resolution of the initial condition or trauma, producing or contributing to the chronicity of orofacial pain [17]. Hypervigilance to pain-related stimuli, borne out of fear of pain, can maintain pain behaviors, especially avoidance [19].

General fear of pain and fear of dental pain, specifically, are associated with greater intensity and duration of acute dental pain [10], and, importantly, fear of pain has been linked to the experience of chronic orofacial pain [9, 20]. In other chronic pain conditions (e.g., low back pain and headache), fear of pain has been associated with poor outcomes such as pain intensity, reduction in physical activity and movement, disability, avoidance, complaining, and maladaptive help-seeking [21–25]. Given that fear of pain may in general be particularly important in the development and maintenance of chronic orofacial pain, research that seeks to more comprehensively characterize fear of pain is critical.

Though fear of pain has been well described and is relatively well researched [14] (see Asmundson et al., 2004 [17], for a review), extant literature primarily addresses environmental, behavioral, and cognitive determinants, with very limited attention given to genetic ones. In the case of anxiety and fear generally, anxiety disorders have been shown to have a moderate-to-high degree of heritability, suggesting that genetic variants influence an individual's risk of developing an anxiety disorder [26–28]. Moreover, a number of specific genes, associated pathways, and gene-environment interactions have been implicated in the development of pathological fear and/or anxiety disorders [29, 30]. Only one previous study, completed by the present research group, has addressed the heritability of fear of pain, with results indicating that such fear is heritable (34%) and shares a genetic basis, in part, with dental care-related fear, a distinct but related phenomenon [31]. These results are consistent with other studies showing that genetic variation contributes to dental care-related fear [32–35].

Given that fear of pain is heritable to some degree, it is likely that genetic variants are etiologically important. Therefore, the first genome-wide association study (GWAS) of fear of pain was performed to identify specific genes potentially driving the heritability of fear of pain. In brief, GWAS involves testing associations between genotype and phenotype for millions of individual single-nucleotide polymorphisms (SNPs; i.e., genetic variants) across the genome to identify the genetic loci related to the phenotype of interest. This preliminary study was completed to provide specific direction for future research that will advance the understanding of fear of pain, which later may offer targets for intervention for patients experiencing orofacial pain acutely, chronically, or both.

## 2. Methods

### 2.1. Sample

Participants were members of families taking part in a large, household-based, cross-sectional study carried out by the Center for Oral Health Research in Appalachia (COHRA1; previously described by Polk and colleagues [36]). The primary aim of the COHRA1 research program was to investigate the multiple factors contributing to the oral health disparities observed in northern Appalachia [36]. Over 800 families with children aged 1–17 years were recruited from West Virginia and western Pennsylvania into the COHRA1 cohort. Participants had dental examinations and provided extensive demographic, medical, and psychosocial data, along with oral microbial samples and DNA for genetic analysis. Participants provided data during one study visit each, which was completed between 2003 and 2009. For analyses presented here, only participants aged 11 years and older were included, given that this was the COHRA1 protocol cut-off age for administration of the fear of pain self-report assessment instrument. All participants provided informed consent, and all study protocols were approved by the Institutional Review Boards of the University of Pittsburgh and West Virginia University.

### 2.2. Fear of Pain Questionnaire-9

The COHRA1 protocol included the Fear of Pain Questionnaire-9 (FPQ-9), a self-report psychological assessment of fear of pain, which tapped the psychological phenotypes used in this GWAS. The FPQ-9 is patterned after its validated parent scale, the Fear of Pain Questionnaire-III (FPQ-III) [37], and comprises three subscales—fear of severe pain, fear of minor pain, and fear of medical/dental pain. Extensive psychometric data are available for the FPQ-III [38, 39]. Each subscale consists of three items that yield psychometrically reliable and validated scores [37]. Total score ranges 9–45, with higher scores indicating greater levels of fear of pain.

The FPQ-9 was developed as a self-report measure of fear of pain to be administered to adults and adolescents; given the reading level of FPQ-9 items, and the nature of their content, administration of the FPQ-9 to younger children is not developmentally appropriate. Thus, in developing the study protocol, and to ensure validity of data collected, a cut-off of 11 years was set for administration (and parent completion was disallowed as the FPQ-9 is an assessment tool designed for adult and adolescent self-report).

### 2.3. Genotyping, Quality Assurance, and Imputation

DNA was extracted from blood or saliva samples (which result in equivalent genotypes [40]) and genotyped for over 560K SNPs using the Illumina Human610-Quad_v1_B BeadChip [41] by the Center for Inherited Disease Research at Johns Hopkins University. The source of DNA (i.e., blood versus saliva) and the method of sample collection were a function of time of enrollment in study and based on optimal DNA extraction methods available at that time [36]; of COHRA1 participants, 43% provided blood samples. Regardless of source of DNA, standard data cleaning procedures were conducted for all genetic data, as previously described [42, 43]. In brief, samples were interrogated for genotype call rate, batch effects, chromosomal aberrations, expected and cryptic relatedness, genetic sex, evidence of sample contamination, and genetic ancestry. SNPs were interrogated for call rate, deviations from Hardy-Weinberg equilibrium, discordance among technical duplicates, and Mendelian errors among biologically related samples. Based on these analyses, quality assurance filters were implemented to omit poorly genotyping samples and SNPs. Imputation of unobserved SNPs was performed as implemented in IMPUTE2 [44, 45] (after prephasing with SHAPEIT2 [46]), utilizing haplotypes from Phase 3 of the 1000 Genomes Project as the reference sample. Imputed dosages were filtered on per-SNP and per-genotype-per-person levels and translated to high-confidence hard calls. Imputation quality filters were applied at the SNP level (i.e., INFO score > 0.5) and genotype per individual level (i.e., genotype probability > 90%). A minor allele frequency filter of 0.01 was applied to both genotyped and imputed SNPs.

### 2.4. Statistical Analysis

Independent-samples* t*-tests were conducted to confirm whether and to what degree sex differences exist in the self-report of fear of pain for this sample, as such differences have been widely reported in the fear literature [47]. Multiple linear regression analyses were conducted to determine relations between FPQ-9 total/subscale scores (dependent variables) and possible covariates (i.e., age, education, income, and sex); FPQ-9 total and subscale score distributions were amenable to these analyses. The threshold for statistical significance of* t*-test and regression analyses was *p* = 0.05.

To simultaneously account for the biological relationships among participants in the COHRA1 cohort as well as any population structure (i.e., genetic variation in the cohort due to ancestry), a variance components modeling framework was utilized for testing the association of each SNP with dental fear, while accounting for the covariates of age, sex, education, and household income as well as genetic sharing. The software EMMAX [48] was used to fit the mixed model. To make the mixed model approach computationally feasible on the genome-scale, the method implemented in EMMAX estimates the variance parameters for the dataset once, rather than separately for each SNP tested. This simplification assumes that any individual SNP has a small effect on the phenotype, which is a safe assumption based on the given results.

GWAS scans were performed for four phenotypes: the FPQ-9 total score and each of the three subscales (minor pain, severe pain, and medical/dental pain). Given the multiple testing burden and the strong linkage disequilibrium (LD; correlation among physically proximal genetic variants due to population history) among SNPs, we used the consensus threshold in the field to declare genome-wide statistical significance at *p* < 5 × 10^−8^. In addition, we interpreted “suggestive” associations in light of any corroborating evidence of relevant biological functions from external data sources. Suggestive associations were defined as one order of magnitude less than genome-wide significance; thus, the threshold for significance in these analyses was *p* < 5 × 10^−7^. The Manhattan and quantile-quantile plots used to visualize the results were generated using the GWASTools package in R (R Foundation for Statistical Computing, Vienna, Austria) [49]. LocusZoom [50] was used to create regional association plots.

## 3. Results

The total number of participants in the COHRA1 dataset aged 11 years and older who completed the FPQ-9 totaled 1,687. Of these, 104 participants were excluded due to missing responses for ≥20% of the FPQ-9 instrument. Of the remaining 1,583 participants, 60 individuals were missing less than 20% of the FPQ-9 responses, which were imputed using mean imputation. Of the 1,583 participants with available phenotype data, complete covariate and genetic data were available for 990, with ages ranging 12–74 years. After cleaning and filtering, a total of 8,591,557 SNPs were available for the GWAS.

Characteristics of the 990 participants included in this study sample are shown in Table 1, with bivariate correlations among demographic variables presented in Table 2. The mean age was 32.2 years (SD = 11.0), and majority of participants were female, which reflects the study design targeting households including parents of minor children. Nearly half of the samples were from households with annual incomes less than $15,000. The majority of the sample had a high-school education or greater; approximately 14% had a college degree or greater.

Results of the independent-samples* t*-tests indicated a mean difference between males and females in general fear of pain, fear of minor pain, fear of severe pain, and fear of medical/dental pain. The mean for females consistently was higher and that of males lower: for general fear of pain, *t*(988) = −7.376  (*p* < 0.001); fear of minor pain, *t*(988) = −3.745  (*p* < 0.001); fear of severe pain, *t*(988) = −7.239  (*p* < 0.001); and fear of medical/dental pain, *t*(988) = −7.013  (*p* < 0.001). Multiple linear regression analyses also were indicative of significant relations between sex, age, education, and household income and fear of pain total and subscale scores, justifying their inclusion in the GWAS analyses as covariates. The results of the regression models are included in Tables 3, 4, 5, and 6.

**Figure 1:**
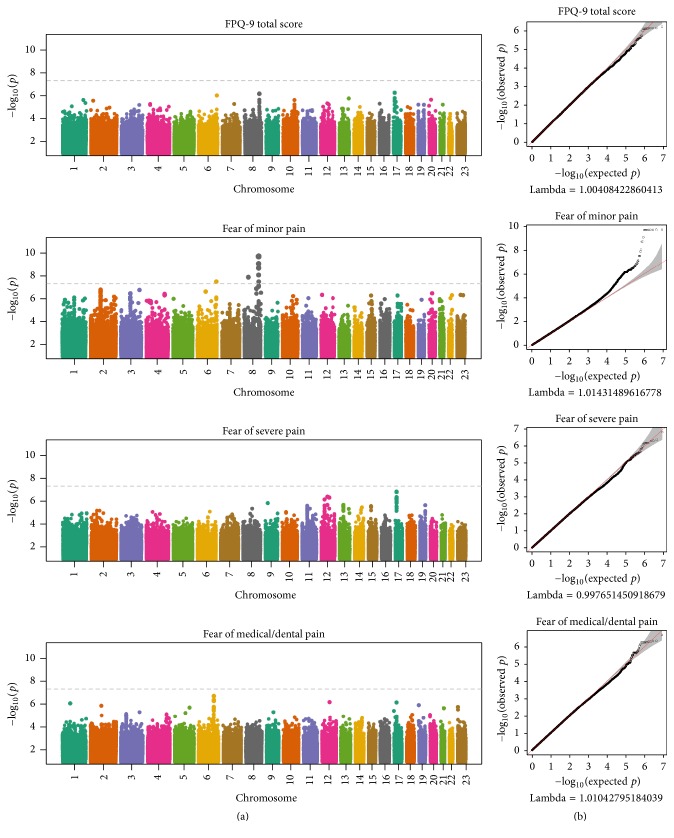
Manhattan plots (a) show the –log_10_-transformed *p* value (*y*-axis), indicating significance of the association, for each SNP across the genome (*x*-axis; organized by physical position on each chromosome). The horizontal dashed lined indicates the threshold for genome-wide significance. Quantile-quantile plots (b) show the –log_10_-transformed *p* values observed in the GWAS (*y*-axis) plotted against the expected distribution of *p* values under the null hypothesis of no association. Chromosome 23 is the X chromosome.

**Figure 2:**
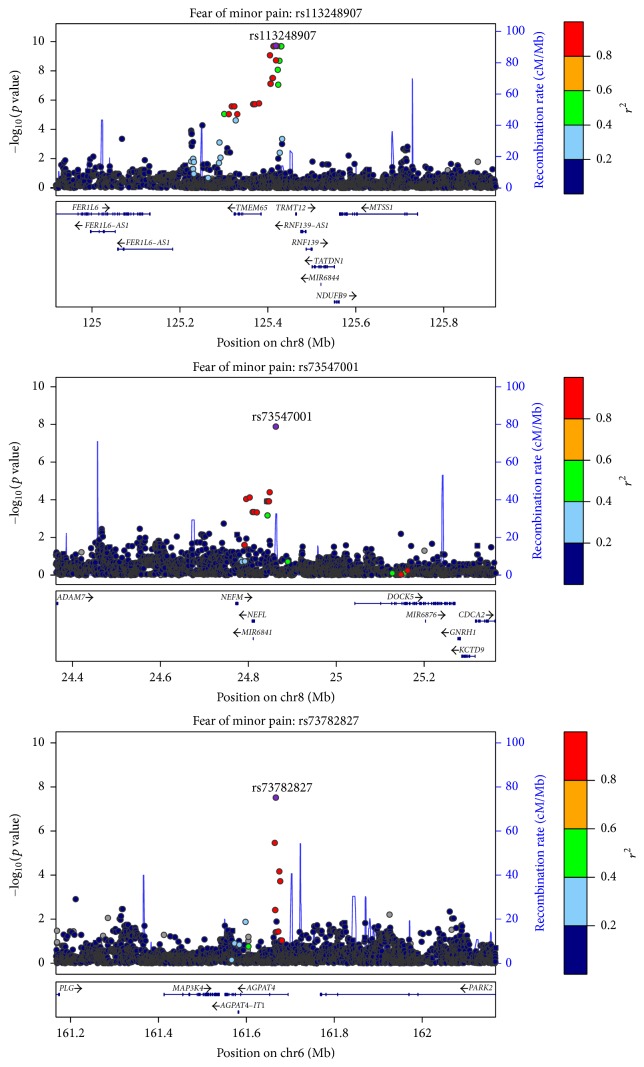
Regional association plots for loci significantly associated with fear of minor pain. Each point depicts the significance (–log_10_-transformed *p* value; *y*-axis) and physical position (Mb; left *x*-axis) of a SNP. The leading SNP is shown as a purple circle and its reference sequence designation is indicated. Open circles represent imputed SNPs, and closed circles represent genotyped SNPs. The coloring of the points represents the LD (i.e., correlation; *r*^2^) of the SNP with the leading SNP. The blue recombination rate overlay (cM/Mb; right *x*-axis) provides information about the LD-block structure of region. Locations of known genes are shown below the plot.

Figure 1 shows the Manhattan and quantile-quantile plots for GWAS scans of the FPQ-9 total score and minor, severe, and medical/dental subscales. The genomic inflation factor ranged from 0.998 to 1.014 across the four scans, indicating no evidence of systematic bias in the behavior of the test statistic. Three genetic loci met the threshold for genome-wide significance (*p* < 5 × 10^−8^) for fear of minor pain. These loci are depicted in the regional association plots shown in Figure 2, and the details for the leading SNPs are shown in Table 7. The most significant association was observed between fear of minor pain and a region on chromosome 8q24.13 (leading SNP rs113248907; *p* = 1.91 × 10^−10^) near the gene* TMEM65*. The leading SNP is intergenic and not in a known regulatory element. Other genome-wide significant associations with fear of minor pain were observed for regions on chromosomes 8p21.2 (leading SNP rs73547001; intronic; *p* = 1.30 × 10^−8^) in* LOC105379330* (an uncharacterized locus that may be a pseudogene or noncoding RNA) near the genes* NEFM* and* NEFL* and 6q26 (leading SNP rs73782827; intronic; *p* = 3.05 × 10^−8^) near the genes* AGPAT4* and* PARK2*. Of note, rs73547001 is in a LOC gene; LOC105379330 is an uncharacterized locus that may be a pseudogene or noncoding RNA.

In addition to the significant associations observed with fear of minor pain, “suggestive” associations were observed across the four GWAS scans. Details for the leading SNP in these regions are shown in Table 7.

## 4. Discussion

Fear of pain is a complex psychological phenomenon that is multifaceted and can contribute to the experience of acute and chronic pain [17]. Fear of pain may be etiologically important in cases of orofacial pain, given the role of emotion in the development and maintenance of orofacial pain problems [14]. In this study, genome-wide association scans were carried out to identify genes that may be related to fear of pain. The objective was to advance the literature suggesting that genetic variation contributes to the etiology of fear of pain by specifying which genes may be implicated.

Three genome-wide significant associations between genetic loci and fear of minor pain were observed, and numerous suggestive associations were observed between genetic loci and general fear of pain (i.e., FPQ-9 total score), fear of minor pain, fear of severe pain, and fear of medical/dental pain (i.e., FPQ-9 subscale scores). Genes near the genome-wide significant loci included* TMEM65*,* NEFM*,* NEFL*,* AGPAT4*, and* PARK2*. Of these genes,* TMEM65* (transmembrane protein 65) is the most interesting in the context of fear of pain.* TMEM65* is among the most differentially methylated in people with chronic widespread musculoskeletal pain [51] and also has been implicated in abnormal pain threshold and predisposition to neuropathic pain in rats [52]. Associations with* NEFM* and* NEFL* also are interesting, as they are associated with neuronal functioning, especially for motor neurons [53, 54] and may be distally related to pain experience.

Perhaps most intriguing is the finding that fear of minor pain was the only phenotype for which significant associations with genetic loci were observed at a genome-wide level. Though suggestive associations were observed for general fear of pain, fear of severe pain, and fear of medical/dental pain, the associations observed for fear of minor pain showed stronger statistical evidence. It may be the case that fear of minor pain differentiates individuals who are particularly pain fearful, with biological underpinnings. To be fearful of severe pain and/or medical/dental pain is perhaps normative, although individuals differ in those regards along a continuum as well [37]. To be phenotypically different in fear of minor pain, however, argues for substantive, perhaps even qualitative individual differences. In the realm of orofacial pain, fearing minor pain may influence “distress tolerance” [55] of even minor dental procedures, seeking of analgesic medications, and delay or avoidance of needed dental care. It is plausible, for instance, that genetic predisposition impacts certain biological pathways associated with sensitivity to low-level pain or distress intolerance, making people more likely to experience and find distressing minor pain, contributing to fear of minor pain [56]. This finding may have implications for understanding the etiological role of fear of pain in the experience of chronic orofacial pain, as it can be characterized by lower-level, annoying background pain.

In carrying out the GWAS to determine which genes may be related to fear of pain, several demographic covariates were controlled for after being analyzed by multiple linear regression, and results from those regression analyses are interesting in their own right. Consistent with previous literature [57, 58], females tended to report higher levels of pain-related fear (generally and across all FPQ-9 subscales) and males lower levels. This trend is observed broadly across fear literature, and researchers have suggested actual sex differences in the experience of many fears/anxieties, reporting biases driven by social norms and/or gender differences in communication style as potentially contributing to the noted difference [47]. In the case of fear of pain, specifically, sex differences in pain perception also may play a role [59, 60]. For general fear of pain, fear of severe pain, and fear of minor pain, age was a significant predictor. Contemporary literature on the effects of age on pain-related fear is mixed and somewhat inconclusive [61]; however, the findings of the current study generally are consistent with previous work suggesting that as an individual's age increases, levels of pain-related fear decrease [62]. It may be that, with age and life experience, people are desensitized to pain-related stimuli such that a reduction in fear is observed [63]. Likewise, for fear of severe pain and fear of minor pain, education was a significant predictor, with greater education associated with lower fear of pain. This finding is consistent with literature demonstrating an association between lower level of education and fear-avoidance beliefs about physical activity in pain patients [64], and it may be that, generally, education can protect people from developing maladaptive or irrational fears [65]. Additional research is needed to fully understand how covariates such as sex, age, and education influence fear of pain.

Some study limitations are noted. First, the study, by design, is cross-sectional in nature with correlational results. Causality cannot be inferred from study results and follow-up studies are necessary to elucidate mechanisms of observed associations. Second, fear of pain data are self-reported. While the instrument used to assess fear of pain is a valid and reliable one, measurement of complex psychological or behavioral phenotypes is stronger when multimodal assessment methods are utilized (e.g., combined use of objective behavioral observation, cognitive assessment or neuroimaging, clinician-rated measures, and/or physiological assessment) [66]. Third, the study sample was recruited from one region of the United States and is relatively homogenous with regard to ethnicity and cultural background, which may bias the experience or reporting of phenotypes like fear of pain, potentially limiting the generalizability of the study. Similar analyses should be completed with other and/or more diverse samples. Lastly, the sample utilized for this study was modest in size for a GWAS, and it is acknowledged that many true associations may have gone undetected due to low power to detect variants of small effect and/or low frequency.

Considering the design of this study, GWAS is understood to be a discovery-driven approach aimed at creating hypotheses. Thus, study results are considered to be preliminary and should guide future research in the area. Replication of the study with other samples is necessary. Moreover, future research should seek to confirm the findings presented here through the use of epidemiological or experimental approaches. Functional studies are needed to determine* how* genetic loci observed to be associated with fear of pain are related to the phenotype. Specific genes suggested by this study to be related to fear of pain should be the subject of future follow-up studies, potentially with attention paid to possible mechanistic pathways that involve pain perception (e.g., hypersensitivity to pain stimuli), anxiety/fear, anxiety sensitivity, and/or distress tolerance. Also, the potentially unique and important role of fear of minor pain in driving general fear of pain and associated pain behaviors and outcomes should be researched more comprehensively. Of note, a recent meta-analysis of GWAS addressing anxiety disorders revealed two genetic loci, rs1709393 and rs1067327, significantly associated with anxiety and/or anxiety disorders, across all published studies [67]. The current dataset was analyzed for associations between variation at these loci and fear of pain, with no evidence found. Future studies should continue to address genetic contributions to anxiety, generally, with attention paid to genetic variation that accounts for both general anxiety and fear of pain, specifically.

This study is one of only a few to address genetic contributions to fear of pain, and it is the first to analyze genome-wide associations to offer avenues for future research. Understanding the role of genetic variation in fear of pain has the potential to improve our understanding of the way emotions affect and are affected by the pain experience. More immediately, discovering specific genetic associations with fear of pain advances a more comprehensive understanding of this complex psychological phenomenon. Given that fear of pain impacts acute orofacial pain and is critically important in its development and maintenance and is a major component of dental care-related anxiety and fear [68], a more completely defined etiology of fear of pain could ultimately refine the management and treatment of orofacial pain by highlighting the best targets for intervention.

## 5. Conclusions

This study aimed to advance the understanding of genetic contributions to fear of pain and was the first GWAS for this psychological phenotype. In carrying out the study, multiple demographic risk factors for higher fear of pain were identified: being female is associated with increased risk of general fear of pain and its components (i.e., fear of medical/dental pain, fear of severe pain, and fear of minor pain); younger age is associated with increased risk of general fear of pain as well as fears of severe and minor pain; and, having less education is associated with increased risk of fears of severe and minor pain. There were three genetic loci identified for fear of minor pain, which may have implications for individuals with lower pain tolerance or with chronic orofacial pain. Findings of this study should guide follow-up genetic studies, including confirmatory ones as well as epidemiological and experimental (e.g., functional or mechanistic) investigations. Understanding genetic and other contributions (such as demographic risk factors) to fear of pain and the specific genes and variants influencing individual differences in fear of pain could lead to innovative ways of managing and mitigating orofacial and other pain, particularly among those who suffer from chronic pain.

## Figures and Tables

**Table 1:** Demographic characteristics of study sample.

	Mean/*N*	Std. deviation/%
Age (years)	32.2	11.0
Sex		
Men	383	38.7%
Women	607	61.3%
Race/ethnicity		
Caucasian	1,367	86.5%
African American	159	10.0%
Hispanic	13	0.8%
Native American/Asian/other	23	1.4%
Missing	21	1.3%
Income		
Less than $10,000 USD	251	25.5%
$10,000 to $14,999	202	20.4%
$15,000 to $24,999	182	18.4%
$25,000 to $34,999	115	11.6%
$35,000 to $49,999	130	13.1%
$50,000 to $74,999	52	5.3%
$75,000 to $99,999	37	3.7%
$100,000 to $149,999	11	1.1%
$150,000 to $199,999	2	0.2%
$200,000 or more	8	0.8%
Education		
No high school diploma	264	26.7%
High school diploma/GED	371	37.5%
Technical school	115	11.6%
Some college, no degree	105	10.6%
Undergraduate degree	84	8.5%
Graduate degree	51	5.2%
Fear of pain		
Total score	23.6	8.1
Fear of minor pain	6.0	2.5
Fear of severe pain	9.8	3.7
Fear of medical/dental pain	7.7	3.3

**Table 2:** Bivariate correlations of covariates used in GWAS model.

	Sex	Age	Education	Income
Sex	1			
Age	−.030	1		
Education	.078^*∗*^	.382^*∗∗*^	1	
Income	−.007	−.011	.006	1

*Note*. *N* = 990. *∗* = correlation is significant at the 0.05 level (2-tailed); *∗∗* = correlation is significant at the 0.01 level (2-tailed).

**Table 3:** Multiple linear regression results for FPQ-9 total score.

Model		*B*	SE	*β*	*t*	*p*	Lower bound	Upper bound
1	(Constant)	19.717	.943		20.911	<0.001	17.866	21.567
	Age	.061	.025	.082	2.461	0.014	.012	.109
	Education	−.091	.187	−.016	−.488	0.626	−.458	.276
	Income	−.104	.131	−.025	−.798	0.425	−.361	.152
	Sex	3.861	.517	.232	7.468	<0.001	2.846	4.875

*Note*. *B* = raw regression coefficient. SE = standard error. *β* = standardized regression coefficient. Sex dichotomized as males = 0; females = 1.

**Table 4:** Multiple linear regression results for FPQ-9 fear of medical/dental pain subscale score.

Model		*B*	SE	*β*	*t*	*p*	Lower bound	Upper bound
1	(Constant)	6.789	.391		17.363	<0.001	6.002	7.557
	Age	.006	.010	.020	.592	0.554	−.014	.026
	Education	−.113	.078	−.049	−1.452	0.147	−.265	.040
	Income	−.015	.054	−.008	−.269	0.788	−.121	.092
	Sex	1.525	.214	.222	7.114	<0.001	1.104	1.946

*Note*. *B* = raw regression coefficient. SE = standard error. *β* = standardized regression coefficient. Sex dichotomized as males = 0; females = 1.

**Table 5:** Multiple linear regression results for FPQ-9 fear of severe pain subscale score.

Model		*B*	SE	*β*	*t*	*p*	Lower bound	Upper bound
1	(Constant)	8.010	.426		18.800	<0.001	7.174	8.846
	Age	.022	.011	.066	1.970	0.049	.000	.044
	Education	.172	.084	.068	2.035	0.042	.006	.338
	Income	−.054	.059	−.028	−.909	0.364	−.170	.062
	Sex	1.665	.234	.221	7.126	<0.001	1.206	2.123

*Note*. *B* = raw regression coefficient. SE = standard error. *β* = standardized regression coefficient. Sex dichotomized as males = 0; females = 1.

**Table 6:** Multiple linear regression results for FPQ-9 fear of minor pain subscale score.

Model		*B*	SE	*β*	*t*	*p*	Lower bound	Upper bound
1	(Constant)	4.917	.298		16.482	<0.001	4.332	5.502
	Age	.033	.008	.142	4.188	<0.001	.017	.048
	Education	−.151	.059	−.087	−2.544	0.011	−.267	−.034
	Income	−.036	.041	−.027	−.870	0.385	−.117	.045
	Sex	.671	.164	.129	4.102	<0.001	.350	.992

*Note*. *B* = raw regression coefficient. SE = standard error. *β* = standardized regression coefficient. Sex dichotomized as males = 0; females = 1.

**Table 7:** Results for the leading SNP for loci showing significant or suggestive evidence of association with fear of pain.

Phenotype	SNP	Chr.	BP	A1	A2	MAF	Type	Info	Beta	*p*
Total	rs73782827	6	161667052	G	T	0.01	Imputed	0.91	−10.84	9.87*E* − 07
	rs112510117	8	125429261	A	G	0.01	Imputed	0.912	−8.44	7.16*E* − 07
	rs7084783	10	105324170	G	A	0.49	Imputed	0.982	−1.69	2.44*E* − 06
	rs56875752	17	12599002	C	A	0.11	Imputed	0.976	2.91	5.94*E* − 07

Minor	rs1146361	1	116658817	C	T	0.15	Imputed	0.994	−0.81	8.12*E* − 07
	rs6542838	2	99452458	C	T	0.44	Imputed	0.997	−0.61	1.56*E* − 07
	rs113865232	2	225213815	T	C	0.01	Imputed	0.986	−2.63	6.74*E* − 07
	rs13411339	2	237231135	T	C	0.03	Imputed	0.961	−1.77	9.12*E* − 07
	rs9871066	3	97202242	G	A	0.02	Imputed	0.993	−2.56	3.44*E* − 07
	rs2699039	4	160353222	A	G	0.02	Imputed	0.993	−2.54	3.74*E* − 07
	rs73746987	6	71820675	T	C	0.01	Imputed	0.974	−3.40	2.26*E* − 07
	rs73782827	6	161667052	G	T	0.01	Imputed	0.91	−3.80	3.05*E* − 08
	rs34139189	6	162293644	G	A	0.14	Imputed	0.796	−0.93	8.22*E* − 07
	rs73547001	8	24862630	G	A	0.01	Imputed	0.89	−3.35	1.30*E* − 08
	rs73700552	8	107068214	C	T	0.01	Imputed	0.969	−3.43	1.30*E* − 07
	rs113248907	8	125418123	A	C	0.01	Imputed	0.95	−3.41	1.91*E* − 10
	rs1871535	8	145639586	C	T	0.02	Imputed	0.878	−2.09	2.91*E* − 07
	rs12252682	10	93810742	C	G	0.02	Imputed	0.852	−2.45	5.89*E* − 07
	rs687735	11	64047152	A	G	0.01	Imputed	0.874	−3.26	8.96*E* − 07
	rs11871659	17	40692638	C	T	0.03	Imputed	0.979	−2.08	5.27*E* − 07
	rs2223785	20	30676316	C	T	0.02	Imputed	0.968	−2.80	3.39*E* − 07
	rs6008192	22	47804243	T	G	0.01	Imputed	0.931	−2.99	4.79*E* − 07
	rs73558352	23	123751759	T	G	0.02	Imputed	0.639	−2.00	4.84*E* − 07

Severe	rs553086	11	56852098	A	G	0.23	Genotyped	1	0.92	2.69*E* − 06
	rs10844026	12	31994740	A	T	0.24	Imputed	0.995	−0.95	7.69*E* − 07
	rs61923480	12	54884202	C	A	0.02	Imputed	0.915	2.52	4.28*E* − 07
	rs61930374	12	71271433	C	G	0.20	Imputed	0.933	−1.09	5.12*E* − 07
	rs112664965	13	51050827	A	G	0.04	Imputed	0.997	−1.88	2.18*E* − 06
	rs7178691	15	53899306	A	G	0.32	Imputed	1	−0.82	2.86*E* − 06
	rs56875752	17	12599002	C	A	0.11	Imputed	0.976	1.38	1.57*E* − 07
	rs3975333	19	53805815	T	A	0.09	Imputed	0.793	1.49	2.29*E* − 06

Medical	rs114134414	1	74708559	C	A	0.03	Imputed	0.899	2.15	9.29*E* − 07
	chr2:99411513	2	99411513	A	T	0.14	Imputed	0.719	−1.29	1.43*E* − 06
	rs72965720	6	128023381	C	A	0.08	Imputed	0.999	1.43	2.06*E* − 07
	rs9901616	17	29336745	T	C	0.03	Imputed	0.895	−2.31	7.69*E* − 07
	rs10422046	19	982968	A	G	0.11	Imputed	0.761	1.38	1.22*E* − 06
	rs5979239	23	10009993	A	G	0.04	Imputed	0.972	−2.04	1.82*E* − 06

*Note.* chr. = chromosome; BP = base position; A1 = minor (effect) allele; A2 = major allele; MAF = minor allele frequency; info = imputation information metric, an indicator imputation quality; beta = effect size per minor allele.
